# Hypermethylation of *TMEM240* predicts poor hormone therapy response and disease progression in breast cancer

**DOI:** 10.1186/s10020-022-00474-9

**Published:** 2022-06-17

**Authors:** Ruo-Kai Lin, Chih-Ming Su, Shih-Yun Lin, Le Thi Anh Thu, Phui-Ly Liew, Jian-Yu Chen, Huey-En Tzeng, Yun-Ru Liu, Tzu-Hao Chang, Cheng-Yang Lee, Chin-Sheng Hung

**Affiliations:** 1 Program in Drug Discovery and Development Industry, Program in Clinical Drug Development of Herbal Medicine, Master Program in Clinical Genomics and Proteomics, College of Pharmacy, Graduate Institute of Pharmacognosy, Taipei Medical University, 250 Wu-Hsing Street, Taipei, Taiwan; 2 Clinical Trial Center, Taipei Medical University Hospital, 252 Wu-Hsing Street, Taipei, Taiwan; 3 Division of General Surgery, Department of Surgery, Shuang Ho Hospital, Taipei Medical University, New Taipei City, Taiwan; 4 Department of Surgery, School of Medicine, College of Medicine, Taipei Medical University, Taipei, Taiwan; 5 Quang Tri Medical College, Dien Bien Phu Str., Dong Luong District, Dong Ha City, Quang Tri Vietnam; 6 Department of Pathology, Shuang Ho Hospital, Taipei Medical University, New Taipei, Taiwan; 7 Department of Pathology, School of Medicine, College of Medicine, Taipei Medical University, Taipei, Taiwan; 8 School of Pharmacy, College of Pharmacy, Taipei Medical University, Taipei, Taiwan; 9 Division of Hematology and Oncology, Department of Medicine, Taipei Medical University Hospital, Taipei, Taiwan; 10 Department of Medical Research, Division of Hematology/Medical Oncology, Department of Medicine, Taichung Veterans General Hospital, Taichung City, Taiwan; 11 Program for Cancer Molecular Biology and Drug Discovery, and Graduate Institute of Cancer Biology and Drug Discovery, College of Medical Science and Technology, Taipei Medical University, Taipei, Taiwan; 12 Joint Biobank, Office of Human Research, Taipei Medical University, Taipei, Taiwan; 13 Graduate Institute of Biomedical Informatics, College of Medical Science and Technology, Taipei Medical University, Taipei, Taiwan; 14 Bioinformatics Center, Office of Data Science, Taipei Medical University, Taipei, Taiwan; 15 Division of General Surgery, Department of Surgery, Taipei Medical University Hospital, Taipei, Taiwan

**Keywords:** TMEM240, Breast cancer, DNA methylation, Circulating cell-free DNA, Disease progression, Tumor suppressor genes, Proliferation, Cell motility, RNA-seq, MetaCore and hormone therapy

## Abstract

**Background:**

Approximately 25% of patients with early-stage breast cancer experience cancer progression throughout the disease course. Alterations in *TMEM240* in breast cancer were identified and investigated to monitor treatment response and disease progression.

**Methods:**

Circulating methylated *TMEM240* in the plasma of breast cancer patients was used to monitor treatment response and disease progression. The Cancer Genome Atlas (TCGA) data in Western countries and Illumina methylation arrays in Taiwanese breast cancer patients were used to identify novel hypermethylated CpG sites and genes related to poor hormone therapy response. Quantitative methylation-specific PCR (QMSP), real-time reverse transcription PCR, and immunohistochemical analyses were performed to measure DNA methylation and mRNA and protein expression levels in 394 samples from Taiwanese and Korean breast cancer patients. *TMEM240* gene manipulation, viability, migration assays, RNA-seq, and MetaCore were performed to determine its biological functions and relationship to hormone drug treatment response in breast cancer cells.

**Results:**

Aberrant methylated *TMEM240* was identified in breast cancer patients with poor hormone therapy response using genome-wide methylation analysis in the Taiwan and TCGA breast cancer cohorts. A cell model showed that TMEM240, which is localized to the cell membrane and cytoplasm, represses breast cancer cell proliferation and migration and regulates the expression levels of enzymes involved in estrone and estradiol metabolism. TMEM240 protein expression was observed in normal breast tissues but was not detected in 88.2% (67/76) of breast tumors and in 90.0% (9/10) of metastatic tumors from breast cancer patients. QMSP revealed that in 54.5% (55/101) of Taiwanese breast cancer patients, the methylation level of *TMEM240* was at least twofold higher in tumor tissues than in matched normal breast tissues. Patients with hypermethylation of TMEM240 had poor 10-year overall survival (*p* = 0.003) and poor treatment response, especially hormone therapy response (*p* < 0.001). Circulating methylated *TMEM240* dramatically and gradually decreased and then diminished in patients without disease progression, whereas it returned and its levels in plasma rose again in patients with disease progression. Prediction of disease progression based on circulating methylated *TMEM240* was found to have 87.5% sensitivity, 93.1% specificity, and 90.2% accuracy.

**Conclusions:**

Hypermethylation of *TMEM240* is a potential biomarker for treatment response and disease progression monitoring in breast cancer.

**Supplementary Information:**

The online version contains supplementary material available at 10.1186/s10020-022-00474-9.

## Background

Breast cancer is the most common cancer in women, surpassing lung cancer, worldwide (Cancer Registry Annual Report 2017;  Ministry of Health and Welfare 2020; Bray et al. 2018). The estrogen-dependent nature of breast cancer is the fundamental basis for hormone therapy. The hormone receptor-positive human epidermal growth factor receptor 2-negative (HR+/HER2–) subtype, which is characterized by the expression of estrogen receptor (ER) and/or progesterone receptor (PR) without HER2 overexpression/amplification, accounts for approximately 70% of breast cancer patients. However, estrogen-independent growth often exists de novo at diagnosis or develops during the course of hormone therapy. Nearly 20–30% of patients with early-stage disease become metastatic throughout the disease course (Zhu and Xu 2021). Therefore, ER expression alone is insufficient in predicting endocrine therapy efficacy (Ma et al. 2016). A significant number of these patients will develop either primary or secondary hormone resistance, prompting the need for tests that can predict treatment response before treatment options are chosen. The initiation and progression of cancer, which is conventionally considered a genetic disease, involve epigenetic abnormalities (Sharma et al. 2010). Genomic screening of 98 different primary human tumors revealed that on average, approximately 600 aberrantly methylated CpG islands exist in each tumor (Costello et al. 2000). Gene silencing by aberrant DNA methylation of promoter and exon regions remains the most dominant phenomenon in breast cancer (Chen et al. 2019; Batra et al. 2021; Jovanovic et al. 2010). The DNA methylation status of *estrogen receptor 1 (ESR1)* and *cytochrome P450 family 1 subfamily B member 1 (CYP1B1)* has been suggested as a marker for treatment response in patients receiving and not receiving tamoxifen as hormonal treatment (Widschwendter et al. 2004).

Combined analysis of data from Taiwanese individuals for whom both data on breast cancer tissue and data on clinical hormone treatment response are available in the TCGA database has shown that hypermethylation of the gene encoding *transmembrane protein gene 240* (*TMEM240*) is a biomarker of poor hormone therapy response in breast cancer. *TMEM240* encodes a transmembrane domain-containing protein found in the brain and cerebellum. In studies of patients from France, Germany, the Netherlands, Colombia, Japan, and China, mutations in *TMEM240* have been found to cause spinocerebellar ataxia 21 (SCA21) with mental retardation, severe cognitive impairment, and hypokinetic and hyperkinetic movement disorders (Delplanque et al. 2014; Traschutz et al. 2019; Yahikozawa et al. 2018; Zeng et al. 2016). The pathogenic mechanism of SCA21 may be mediated through the induction of early gliosis and lysosomal impairment by mutant *TMEM240* (Seki et al. 2018). Hypermethylation of *TMEM240* has been found in colorectal cancer (CRC) (Chang et al. 2020; Naumov et al. 2013). Few reports are available regarding *TMEM240* in women with cancer, and the role of *TMEM240* in breast cancer remains unclear.

Advances in detection technology have reduced breast cancer death rates in several Western countries (DeSantis et al. 2019). Therefore, developing biomarkers for treatment response can improve patient outcomes. For current disease progression monitoring for breast cancer patients, simultaneous use of the two serum markers CA-153 and carcinoembryonic antigen (CEA) shows that the early diagnosis of metastasis in up to 60–80% of patients with breast cancer is not sensitive enough to monitor disease progression in real time (Dawson et al. 2013; Duffy et al. 2010; Banin Hirata et al. 2014). Therefore, no dynamic monitoring system for accurately and sensitively measuring recurrence or metastasis events is available in current clinical practice. Circulating cell-free DNA (ccfDNA) in plasma can be used for the noninvasive sampling of cancer cells obtained from patients with breast cancer (Li et al. 2016). Cells release cell-free DNA through a combination of apoptosis, necrosis, and active secretion. Cancer cells, as well as cells in the tumor microenvironment, can produce ccfDNA. Multiple genetic and epigenetic alterations are found in ccfDNA (Wan et al. 2017). Assays of circulating methylated DNA (cmDNA) could be used for outcome prediction in metastatic breast cancer patients treated with chemotherapy and/or multikinase inhibitors (Amatu et al. 2019). Circulating methylated *TMEM240* can be successfully detected in patients with CRC (Chang et al. 2020). In this study, the promoter methylation level, the expression level and the biological functions of *TMEM240* will be clarified. Whether circulating methylated *TMEM240* can be detected in blood from individuals with breast cancer and its association with treatment response and disease progression will also be investigated.

## Methods

### Patients and tissue, plasma collection

A total of 335 Taiwanese clinical samples, including 137 human breast tumor tissues, 137 adjacent normal breast tissue samples and 61 plasma samples, were obtained from Taipei Medical University (TMU) Hospital, Shuang Ho Hospital and the TMU Joint Biobank. Three sets of tissue microarrays of breast cancer tissues were performed to analyze TMEM240 protein expression. Two sets of microarrays were performed in the Department of Pathology of Shuang Ho Hospital. The tissue microarrays contained breast tumor tissues and matched adjacent normal breast tissues obtained from 36 Taiwanese breast cancer cases. Three tissue microarrays representing a total of 131 tissues, including 76 breast carcinoma tissues, 10 matched metastatic carcinoma tissues and 45 matched normal tissues from South Korea, were purchased from SuperBioChips Laboratories (catalog number CBA4; South Korea), and tissues from Taiwanese breast cancer cases were obtained from the Department of Pathology, Shuang Ho Hospital (Taiwan). The pathologic diagnoses of these cases were microscopically confirmed by two researchers. Prior to the collection of clinical data and samples, written informed consent was obtained from all patients. Patients undergoing preoperative chemoradiotherapy or an emergent operative procedure were excluded from this study. Sections of cancerous tissue and corresponding noncancerous tissues were reviewed by a senior pathologist. Clinical data regarding age, sex, tumor type, TNM tumor stage, menopausal state, estrogen receptor (ER), progesterone receptor (PR), and human epidermal growth factor receptor 2 (HER2) tumor markers, were prospectively collected and obtained from Taipei Medical University (TMU) Hospital, Shuang Ho Hospital and the TMU Joint Biobank. Following surgery, patients were monitored every 3 months for the first 2 years and semiannually thereafter.

### Genomic DNA, circulating cell-free DNA and RNA extraction

Genomic DNA from matched pairs of primary tumors and adjacent breast tissues was extracted using the QIAamp DNA Mini Kit (Qiagen, Bonn, Germany, Cat. No. 51306) according to the manufacturer’s instructions. The tumor and normal specimens that were used for RNA extraction were frozen immediately after surgical resection and stored at − 80 °C. Total mRNA was extracted using the RNeasy Plus Mini Kit (Qiagen, Hilden, Germany, Cat. No. 74134) according to the manufacturer’s instructions. Circulating cell-free DNA was extracted from plasma (3.5 mL) that had been isolated from 10 mL of peripheral blood within 2 h of collection. Circulating cell-free DNA (ccfDNA) was extracted from 15 of the plasma samples using the MagMAX Cell-Free DNA Isolation Kit (Thermo Fisher Scientific, Austin, TX, USA) according to the manufacturer’s recommended protocol (Yan et al. 2018; Hung et al. 2018; Wang et al. 2021). CcfDNA was extracted from 46 of the plasma samples using the iCatcher Circulating cfDNA 1000 kit (CatchGene, New Taipei City, Taiwan) according to the manufacturer’s recommended protocol.

### Reverse transcription PCR

To measure *TMEM240* mRNA expression, real-time reverse transcription PCR (RT–PCR) was performed in a LightCycler 96 (Roche Applied Science, Penzberg, Germany). Real-time PCR was performed using the SensiFAST™ Probe No-ROX Kit (Bioline, London, UK, Cat. No. BIO-86020) with specific primers and the corresponding Universal Probe Library probe (Roche Applied Science, Mannheim, Germany) according to the manufacturer’s instructions. The glyceraldehyde 3-phosphate dehydrogenase gene (GAPDH) was used as a reference gene. The PCR conditions were as follows: preincubation at 95 °C for 10 min followed by 40 cycles of amplification at 95 °C for 10 s and 60 °C for 10 s. The normalized gene expression values obtained using LightCycler Relative Quantification software (Version 1.5, Roche Applied Science) were compared with those of the control group. *TMEM240* mRNA expression was considered low if the mRNA expression level of *TMEM240* relative to *GAPDH* was 0.5-fold lower in the breast tumor tissue than in the paired normal breast tissue. The primers and probes used in RT–PCR are listed in Additional file 1: Table S1.

### TaqMan quantitative methylation-specific PCR

After bisulfite conversion of DNA using the EpiTect Fast DNA Bisulfite Kit (Qiagen, Bonn, Germany, Cat. No. 59826), the DNA methylation level of *TMEM240* was measured using TaqMan quantitative methylation-specific PCR (QMSP) in a LightCycler 96 (Roche Applied Science, Penzberg, Germany). QMSP was performed using the SensiFAST™ Probe No-ROX Kit (Bioline, London, UK, Cat. No. BIO-86020) with specific primers and methyl-TaqMan probe for *TMEM240*. Normalized DNA methylation values, which were calibrated to the control group, were obtained using LightCycler Relative Quantification software (Version 1.5, Roche Applied Science). The *beta-actin* (*ACTB*) gene was used as a reference gene. Primers and probes for *TMEM240* methylation detection were designed to bind to the junction between the promoter and exon 1. The QMSP conditions were as follows: preincubation at 95 °C for 10 min followed by 50 cycles of amplification at 95 °C for 10 s and 60 °C for 10 s. *TMEM240* was considered hypermethylated when the methylation level of *TMEM240* relative to that of the ACTB gene was at least twofold higher in the breast tumor than in the paired normal breast tissue sample. The specificity of *TMEM240* methylation end products was confirmed by bisulfite sequencing (Additional file 1: Fig. S1). The primers and probes used in QMSP are listed in Additional file 1: Table S1.

### Genome-wide methylation analysis

Genome-wide methylation analysis of 5 paired breast cancer tissues and corresponding noncancerous breast tissues was performed using the Illumina Infinium HumanMethylation450 BeadChip array (Illumina, San Diego, CA, USA) for one sample and the Infinium MethylationEPIC Kit (Illumina) for the remaining 4 samples, as previously reported (Chang et al. 2020). The two arrays contain more than 450,000 and 850,000 methylation sites, respectively, and provide genome-wide coverage of the gene region and CpG island coverage, respectively, including 99% of RefSeq genes. Bisulfite conversion of 500 ng of genomic DNA was performed using the EpiTect Fast DNA Bisulfite Kit (Qiagen, Bonn, Germany, Cat. No. 59826). Methylation scores for each CpG site were represented as “beta” values ranging from 0 (unmethylated) to 1 (fully methylated) based on determination of the ratios of the methylated signal intensities to the sums of the methylated and unmethylated signal outputs.

### Cell lines, cell culture, and drug treatment

The MDA-MB-231 and T47D breast cancer cell lines used in this study were obtained from the Bioresource Collection and Research Center (http://www.bcrc.firdi.org.tw/). MDA-MB-231 cells were cultured in DMEM/F12 supplemented with human platelet lysate (hPL, American Red Cross, USA) and 1% penicillin/streptomycin. T47D cells were cultured in DMEM/F12 supplemented with human platelet lysate (hPL, American Red Cross, USA), 1% penicillin/streptomycin and 6 ng/ml insulin. For the *TMEM240* demethylation assay, MDA-MB-231 cells were treated with dimethyl sulfoxide (DMSO) or with the demethylation agent decitabine (DAC, Sigma–Aldrich, St. Louis, MO, USA) for 96 h. DAC was dissolved in DMSO. After treatment of the cells, DNA and RNA were extracted, and methylation and gene expression levels were analyzed. For the hormone therapy response assay, T47D cells were treated with DMSO or with a series of concentrations of Tamoxifen (0, 10 and 20 μM) for 48 h (Sigma–Aldrich, St. Louis, MO, USA).

### RNA sequencing and pathways and networks analysis

Total mRNA was extracted from MDA-MB231 breast cancer cells transfected with vector control or TMEM240 exogenous expression using the RNeasy Plus Mini Kit (Qiagen, Hilden, Germany, Cat. No. 74134) according to the manufacturer’s instructions. The purified RNA was used for the preparation of the sequencing library by the TruSeq Stranded mRNA Library Prep Kit (Illumina, San Diego, CA, USA) following the manufacturer’s recommendations. Briefly, mRNA was purified from total RNA (1 μg) by oligo(dT)-coupled magnetic beads and fragmented into small pieces under elevated temperature. First-strand cDNA was synthesized using reverse transcriptase and random primers. After the generation of double-strand cDNA and adenylation on the 3’ ends of DNA fragments, the adaptors were ligated and purified with an AMPure XP system (Beckman Coulter, Beverly, USA). The quality of the libraries was assessed on the Agilent Bioanalyzer 2100 system and a real-time PCR system. The qualified libraries were then sequenced on an Illumina NovaSeq 6000 platform with 150 bp paired-end reads generated by Genomics, BioSci & Tech Co., New Taipei City, Taiwan. TMEM240-involved pathways and networks in cancer were analyzed by the MetaCore repository (Clarivate Analytics, Philadelphia, USA) (Arai et al. 2014).

### Immunofluorescence assay

For immunofluorescence staining assays, cells were seeded in 4-well glass chamber slides (Nunc). After TMEM240 plasmid overexpression, the DLD-1 cells were fixed in 4% formaldehyde and stained with anti-DDK (1:200, Abcam, Cambridge, UK). Imaging was performed using deconvolution fluorescence microscopy (Olympus).

### Immunohistochemical assay

Immunohistochemical staining with an antibody against TMEM240 (1:35, Sigma–Aldrich, HPA066721, St. Louis, MO, USA) was performed using an iView DAB detection kit (Ventana, Tucson, AZ, USA) and a BenchMark XT autostainer. The assay included both positive and negative controls. The researchers who evaluated the immunohistochemical staining results were blinded to the clinical follow-up data. The intensity of TMEM240 expression was identified semiquantitatively as no expression, low expression (weaker than or equal to the expression intensity observed in normal colon epithelium), or high expression (stronger than the expression intensity observed in normal colon epithelium).

### Plasmid extraction, confirmation and purification

Plasmid DNA was extracted using the Geneaid™ Midi Plasmid Kit (Geneaid Biotech Ltd., Cat. No. PI025) according to the manufacturer’s instructions. The extracted DNA was subjected to preliminary length analysis by sequenced to confirm errorless production. The plasmid concentration was measured using a NanoDrop 2000C ultramicrowavelength spectrometer (Thermo Fisher Scientific, USA), and the plasmid was stored at − 20 °C until further use.

### cDNA expression construct, RNAi, and transfection

*TMEM240* interference RNA was obtained from Life Technologies Corporation. Transfections were performed using 10 nM si-*TMEM240* or nontargeting siRNAs, and Lipofectamine-RNAiMax and Lipofectamine 3000 reagent (Invitrogen) was used to transfect MDA-MB-231 and T47D cells according to the manufacturer's protocol.

### Transwell assay

Transwell assays were used to study cell migration. In the transwell assays, the upper and lower chambers of the culture wells were separated by a semipermeable membrane (Falcon) with a pore size of 8 μm. Approximately 2 × 10^4^ and 1 × 10^5^ treated and untreated MDA-MB-231 and T47D cells, respectively, were seeded in the upper chamber. Then, 300 μL of serum-free DMEM/F12 was added as culture medium, and 800 μL of serum-containing culture medium was added as a chemical attractant in the lower chamber. After 16 h of incubation, the cells retained over the membrane were washed twice with PBS, fixed with 4% formaldehyde and stained with 1% crystal violet/ddH_2_O for 60 min at room temperature. Five randomly chosen areas were photographed using a camera attached to a microscope (Nikon), and ImageJ was used to quantify the number of cells in each area.

### Wound healing assay

The wound healing assays were performed using culture inserts (Ibidi, GmbH, Martinsried, Germany). After seeding 1 × 10^5^ cells overnight, the cells were transfected with siRNA for 48 h. The culture inserts were then removed, the wounded areas were photographed using a camera attached to a microscope (Nikon), and ImageJ was used to calculate the wound areas.

### Sulforhodamine B assay

A sulforhodamine B (SRB) assay was used to measure the cell proliferation rate. MDA-MB-231 and T47D cells were seeded in 96-well plates at densities of 8 × 10^3^ and 1 × 10^4^ cells/well, respectively, and incubated for 24 h and 48 h, respectively. The cells were then fixed with 10% trichloroacetic acid for 10 min. After staining with SRB for 30 min, excess dye was removed by washing the cells five times with 1% acetic acid. Cell proliferation was assessed using a microplate reader to determine the absorbance of the SRB solution at 515 nm.

### The cancer genome atlas portal and data analysis

The data of the Western cohort are based on data generated by The Cancer Genome Atlas (TCGA) Research Network from the Genomic Data Commons (GDC) data portal (https://portal.gdc.cancer.gov/) and were downloaded between 2015 and 2021. For DNA methylation analysis, Illumina Infinium HumanMethylation450 BeadChip array data were analyzed from 582 breast tumors, 87 paired breast cancer and noncancer tissues; 8 tumors from patients with disease progression under hormone therapy and 21 tumors from patients with complete response under hormone therapy; 319 normal tissues including breast, colon, rectal, lung, uterine, gastric, esophagus, pancreases, liver, and prostate adjacent normal tissues. For RNA analysis, RNA-seq data were obtained from 714 breast tumors, 72 paired tumors and normal tissues from breast cancer patients. The copy number variation (CNV) data of 978 paired tumor tissues and peripheral blood cells of breast cancer patients were collected from The Cancer Genome Atlas (TCGA) Breast Invasive Carcinoma Project. We collected CNV data from 978 paired tumor tissues and normal blood cells of breast cancer patients in TCGA databases. Bedtools was used to filter out the germline CNV segments in peripheral blood cells from CNV segments in breast tumor tissues. The somatic CNV-related genes were obtained using GISTIC2.0 analysis (Mermel et al. 2021).

### Statistical analysis

All statistical analyses were performed using SPSS (SPSS Inc., Chicago, IL, USA). Pearson's chi-squared test was used to compare breast cancer patients with respect to *TMEM240* methylation, RNA expression, and clinical data including age, sex, tumor type, TNM tumor stage, race, menopausal state, and ER, PR and HER2 status. The t-test was used to compare cells transfected with or without *TMEM240* plasmid or si-*TMEM240* and cells with and without drug treatment. Multivariate Cox proportional hazards regression analyses (adjusted for age, sex, race, tumor subtype, and tumor stage) were further used to analyze the correlation between *TMEM240* hypermethylation and 10-year overall survival in breast cancer patients. Comparisons of hypermethylation and hypomethylation curves that yielded log-rank test *p* values of less than 0.05 were considered statistically significant. The *TMEM240* methylation level and drug treatment response in breast cancer patients whose data were accessed through the TCGA portal were analyzed using the Mann–Whitney test. In addition to accuracy, other commonly used measures of evaluating the classification, such as the receiver operating characteristic curve (ROC) and area under the curve (AUC), sensitivity, specificity, false-positive rate and false-negative rate, are also reported.

## Results

### *TMEM240* was identified in samples from Taiwanese and Western breast cancer patients by genome-wide methylation analysis

**Fig. 1:**
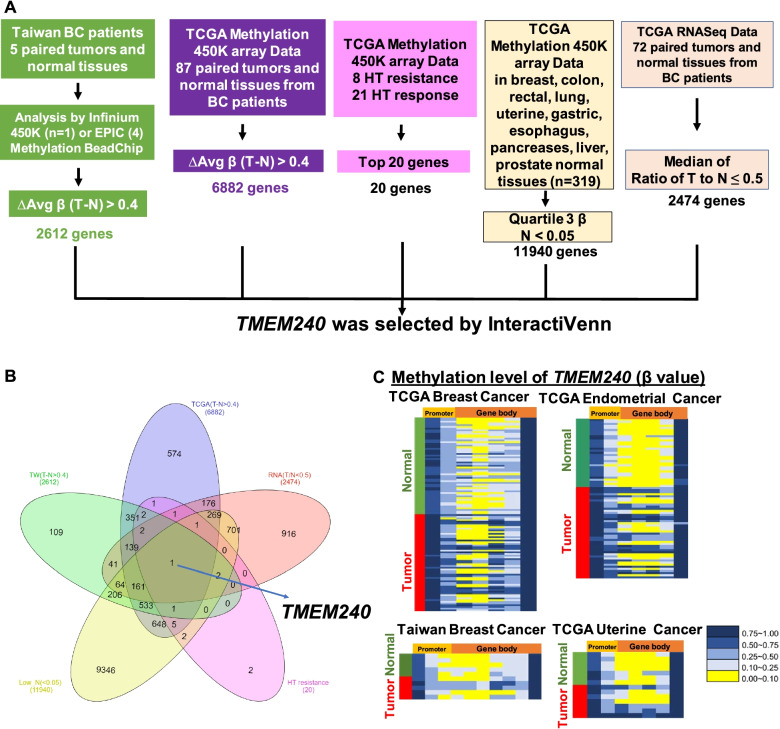
Flowchart of gene selection and heatmap for *TMEM240*. **A** The criteria and step-by-step flowchart for gene selection. **B** Screening of intersecting genes by InteractiVenn. **C** Heatmap of the *TMEM240* methylation pattern in breast cancer and endometrial and uterine cancer

To identify a novel potential biomarker in breast cancer patients with poor hormone therapy response, we used five criteria to screen potential targets: (1) hypermethylation in Taiwanese breast cancer patients; (2) hypermethylation in Western breast cancer patients; (3) hypermethylation in breast cancer patients with poor hormone therapy response compared with complete response; (4) a methylation level in normal tissues that was close to 0; and (5) low expression in breast cancer patients (Fig. 1A). First, to identify critical tumor suppressor genes, the Infinium Methylation Assay was applied to 5 breast cancer tissue samples and paired noncancerous breast tissue samples. A total of 2612 genes were hypermethylated according to the criterion ΔAvg_β (βTumor – βNormal) > 0.4. Second, we analyzed the TCGA Illumina Infinium HumanMethylation450 BeadChip array data of 87 paired Western breast cancer patients. A total of 6882 genes were found to be hypermethylated according to the criterion ΔAvg_β (βTumor – βNormal) > 0.4. Next, the top 20 genes with the highest methylation levels in 8 breast cancer patients with poor hormone therapy response compared with 21 patients with complete response to hormone therapy were identified. Next, we further found that 11,940 genes showed much lower DNA methylation levels in breast, colon, rectal, lung, uterine, gastric, esophageal, pancreatic, liver, and prostate normal tissues. Finally, TCGA RNA sequencing data of 38 paired breast cancer samples from Western patients showed that the expression of 2474 genes were decreased by 50% in the breast cancer samples. The *TMEM240* gene was identified using InteractiVenn (Fig. 1B). Copy number variations (CNVs) could also be a potential biomarker for cancer diagnosis (Pan et al. 2019). Therefore, the CNVs of *TMEM240* were investigated in breast cancer. However, GISTIC2.0 analysis found no local distribution of CNVs in the *TMEM240* gene in breast tumors from patients (Additional file 1: Fig. S2).

Few reports about TMEM240 in women cancer was found. Methylation of *TMEM240* was further analyzed in the TCGA cohort, and the gene was found to be highly methylated in breast cancer, endometrial and uterine cancer. The cluster analysis of the *TMEM240* methylation pattern was visualized as a heatmap (Fig. 1C). The role of TMEM240 in breast cancer is unclear. Therefore, TMEM240 in breast cancer was selected for further analysis. A comprehensive analysis of its epigenetic alterations, mRNA and protein expression was performed, and the biological role of *TMEM240* was further studied.

### TMEM240 represses breast cancer cell proliferation and cell migratory ability

**Fig. 2:**
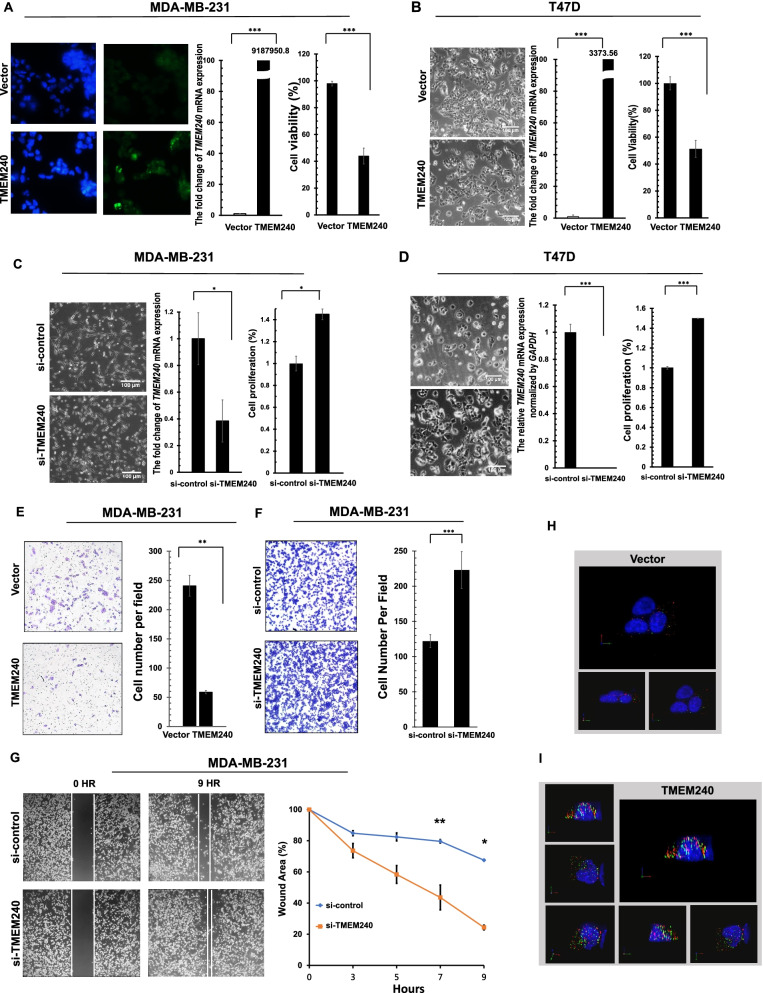
TMEM240 is localized in the cytoplasm and membrane and represses cancer cell growth and migration in breast cancer cells. A recombinant pMyc-DDK-h*TMEM240* plasmid was transfected into MDA-MB-231 breast cancer cells (**A**) and T47D breast cancer cells (**B**) for 24 h, and the cells were then analyzed via immunofluorescence for TMEM240 protein (left, original magnification, × 200) and real-time RT–PCR for mRNA expression (middle). The proliferation of the MDA-MB-231 and T47D cells was analyzed using sulforhodamine B (SRB) assays (right). si-*TMEM240* was transfected into MDA-MB-231 cells (**C**) and T47D cells (**D**). The cell morphology (left, original magnification, × 100), mRNA expression (middle), and rate of cell proliferation (right) of the breast cancer cells were analyzed. **E** The migratory ability of MDA-MB-231 cells after *TMEM240* overexpression was measured via transwell assays. si-*TMEM240* was transfected into MDA-MB-231 cells for 24 h, and the distribution of the cells was then analyzed using transwell assays (**F**) and wound healing assays (**G**). The data are presented as the mean ± SD; *p ≤ 0.05, **p ≤ 0.01, ***p ≤ 0.001. A t-test was used to calculate group differences in all experiments. Experiments were performed using at least two biological duplicates and three technical replicates. Localization of the TMEM240 protein was determined by deconvolution and 3D reconstruction. **H** Recombinant pMyc-DDK vector control. **I** The recombinant pMyc-DDK-h*TMEM240* plasmid was transfected into the cells for 24 h. Red, anti-Myc-hTMEM240 protein. Green, anti-DDK-hTMEM240 protein. Blue, DAPI staining

Alterations in *TMEM240* and its functional roles during tumorigenesis have not been studied previously. To study the biological roles of the TMEM240 protein in breast cancer cells, TMEM240 was overexpressed or knocked down in MDA-MB-231 cells by electroporation. The gene manipulation efficiency was determined through real-time RT–PCR. Transfection of the TMEM240 plasmid into MDA-MB-231 and T47D cells resulted in abundant TMEM240 protein expression (Fig. 2A, left panel) and mRNA expression (Fig. 2A, B, middle panel). According to the SRB cell viability assay, TMEM240 inhibited MDA-MB-231 and T47D cancer cell growth by 55.2% and 48.7%, respectively (Fig. 2A, B, right panel). Microscopic observation revealed that TMEM240 overexpression repressed the growth of T47D and MDA-MB-231 cells compared with a vector control (Fig. 2B, left panel; and Additional file 1: Fig. S3). To determine whether decreased TMEM240 expression induces cell growth, *TMEM240* gene expression was knocked down in MDA-MB-231 and T47D cells using si-*TMEM240*. *TMEM240* mRNA expression was reduced after transfection of the MDA-MB-231 and T47D cells with si-*TMEM240* for 24 h compared with the ci-control (Fig. 2C, D). Microscopic observation and SRB assay revealed that si-*TMEM240* induced proliferation of MDA-MB-231 and T47D cells compared with the si-control group. TMEM240 knockdown increased MDA-MB-231 and T47D cell proliferation by 1.4- and 1.5-fold, respectively (Fig. 2C, D).

To investigate whether *TMEM240* is associated with breast cancer cell migration, MDA-MB-231 cells were transfected with *TMEM240* or si-*TMEM240* for 24 h. The motility of the cells was then analyzed using transwell assays and wound healing assays. The data revealed that an increase in *TMEM240* expression suppressed the migration ability of MDA-MB-231 cells by 75.6% (Fig. 2E). Knockdown of *TMEM-240* in MDA-MB-231 cells significantly induced cell migration by 63.0% and 62.7% based on the results obtained using transwell assays (Fig. 2F) and wound healing assays (Fig. 2G), respectively.

### TMEM240 protein is mainly distributed in cell membranes and in the cytoplasm

Although TMEM240 is predicted to be a membrane protein, to date no study has reported the intracellular distribution of the TMEM240 protein. According to the structure of TMEM240 protein reported in the UniProtKB/Swiss-Prot database, the protein contains two transmembrane protein regions located between amino acid residues 5–25 and 90–110. Exogenous expression of TMEM240 and immunofluorescent staining were used to examine the distribution of the protein in the DLD-1 cell line. Deconvolution and 3D reconstruction of immunofluorescence images showed that the TMEM240 protein was mainly concentrated in the cytoplasm and cell membranes (Fig. 2I).

### Low TMEM240 protein expression in Taiwanese and South Korean breast cancer patients

**Fig. 3:**
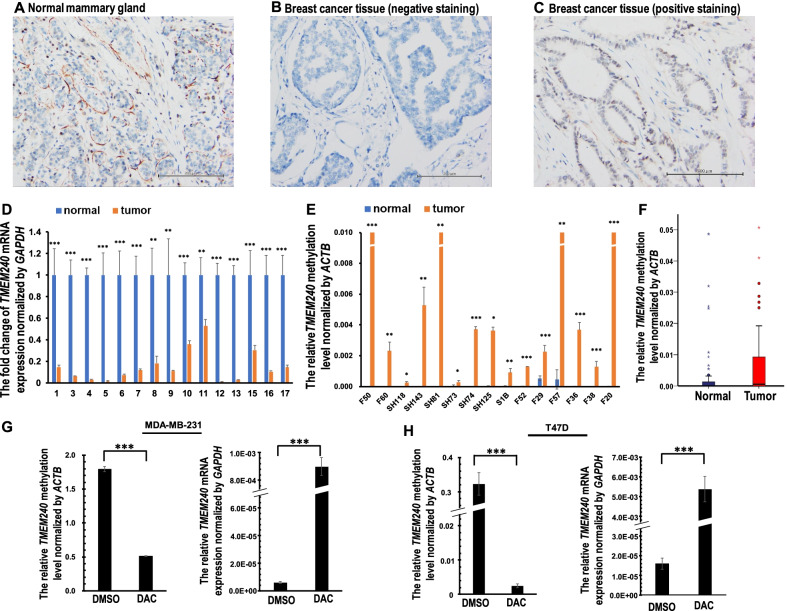
Low expression of TMEM240 in breast cancer is mediated by promoter methylation. Representative figures showing TMEM240 protein expression as analyzed by IHC. **A** Normal mammary gland. **B** Breast cancer tissue with negative expression. **C** Breast cancer tissue with normal expression (original magnification, × 200). The scale bars indicate 200 µm. **D**, **E** Representative figure showing the *TMEM240* mRNA expression level (**D**) and *TMEM240* promoter hypermethylation as determined by RT–qPCR (E) in adjacent breast normal and tumor tissues. **F** Box plot of *TMEM240* promoter hypermethylation levels in tissues. **G**, **H** DNA methylation and mRNA expression were measured after treatment with decitabine (DAC) in MDA-MB-231 (**G**) and T47D (**H**) breast cancer cells. The relative DNA methylation levels after treatment with DAC are shown in the left panel. The relative mRNA expression levels after treatment with DAC are shown in the right panel. The data are presented as the mean ± SD; **p ≤ 0.01, ***p ≤ 0.001. A t-test was used to calculate group differences in all experiments. The experiments were performed using at least two biological duplicates and three technical replicates

TMEM240 reveals the tumor suppressor potential for breast cancer cell growth and migration (Fig. 2). To investigate whether TMEM240 protein expression is altered in cancerous breast tissues, TMEM240 protein expression in 76 breast tumors from 40 Korean breast cancer patients and 36 Taiwanese breast cancer patients was analyzed by immunohistochemistry. TMEM240 was observed to be localized to the cell membrane and cytoplasm in normal breast tissues (Fig. 3A). The protein was expressed at lower than normal levels in 88.2% (67/76) of the tumors from breast cancer patients and in 90.0% (9/10) of metastatic tumors from breast cancer patients (Fig. 3B, C; Table 1). Almost all triple-negative breast cancer patients (95.7%, 22/23) had deficient TMEM240 protein expression (Table 1).

**Table 1:** TMEM240 protein, mRNA expression and promoter hypermethylation in relation to the clinical parameters of Taiwan breast cancer^a^

Characteristics	All^b^	Protein expression		All	mRNA expression^c^				All	DNA methylation^d^			
		Low n (%)	High n (%)		Low n (%)		High n (%)			Low n (%)		High n (%)	
Overall	76	67 (88.2)	9 (11.8)	52	26 (50.0)		26 (50.0)		101	46 (8.9)		55 (54.5)	
Tumor type													
IDC	60	52 (86.7)	8 (13.3)	52	26 (50.0)		26 (50.0)		98	43 (43.9)		55 (56.1)	
ILC	1	1 (100.0)	0 (0.0)	0	0 (0.0)		0 (0.0)		2	2 (100.0)		0 (0.0)	
Others	5	5 (100.0)	0 (0.0)	0	0 (0.0)		0 (0.0)		1	1 (100.0)		0 (0.0)	
Tumor stage													
0, I and II	48	41 (85.4)	7 (14.6)	9	5 (55.6)		4 (44.4)		19	7 (36.8)		12 (63.2)	
III and IV	18	17 (94.4)	1 (5.6)	41	21 (51.2)		20 (48.8)		79	38 (48.1)		41 (51.9)	
Tumor size													
T0–T1	11	10 (90.9)	1 (9.1)	40	24 (60.0)		16 (40.0)^0.040^		30	12 (40.0)		28 (60.0)	
T2–T4	52	46 (88.5)	6 (11.5)	9	2 (22.2)		7 (77.8)		68	33 (48.5)		35 (51.5)	
Lymph node regional metastasis													
No	27	25 (92.6)	2 (7.4)	23	14 (60.9)		9 (39.1)		43	21 (48.8)		22 (51.2)	
Yes	36	31 (86.1)	5 (13.9)	25	11 (44.0)		14 (56.0)		51	24 (47.1)		27 (52.9)	
Grade													
Well	4	3 (75.0)	1 (25.0)	7	3 (42.9)		4 (57.1) ^7^		10	5 (50.0)		5 (50.0)	
Moderate	27	22 (81.5)	5 (18.5)	17	9 (52.9)		8 (47.1)		40	17 (42.5)		23 (57.5)	
Poor	29	27 (93.1)	2 (6.9)	24	13 (54.2)		11 (45.8)		42	20 (47.6)		22 (52.4)	
ER													
Negative	39	35 (89.7)	4 (10.0)	15	8 (53.3)		7 (46.7)		31	14 (45.2)		17 (54.8)	
Positive	37	32 (86.5)	5 (13.5)	27	13 (48.1)		14 (51.9)		68	30 (44.1)		38 (55.9)	
PR													
Negative	42	39 (92.9)	3 (7.1)	16	9 (56.3)		7 (43.8)		40	16 (40.0)		24 (60.0)	
Positive	34	28 (82.4)	6 (17.6)	26	12 (46.2)		14 (53.8)		59	28 (47.5)		31 (52.5)	
HER2													
Negative	42	51 (92.7)	4 (7.3)^0.046^	18	10 (55.6)		8 (44.4)		39	18 (46.2)		21 (53.8)	
Positive	21	16 (76.2)	5 (23.8)	22	20 (45.5)		3 (54.5)		59	25 (42.4)		34 (57.6)	
TNBC													
Yes	23	22 (95.7)	1 (4.3)	10	5 (50.0)		5 (50.0)		15	6 (40.0)		9 (60.0)	
No	53	45 (84.9)	8 (15.1)	32	16 (50.0)		16 (50.0)		84	38 (45.2)		46 (54.8)	
Ki-67													
< 14%	10	9 (90.0)	1 (10.0)	11	5 (45.5)		6 (54.5)		26	13 (50.0)		13 (50.0)	
≧14%	16	11 (68.8)	5 (31.3)	23	10 (43.5)		13 (56.5)		66	27 (40.9)		39 (59.1)	
p53													
Negative	26	26 (100.0)	0 (0.0)										
Positive		22 (88.0)	3 (12.0)										

^a^These results were analyzed by the Pearson *X*^*2*^ test. *P* values with significance are shown as superscripts

^b^For some categories, the number of samples (n) was lower than the overall number analyzed because clinical data were unavailable for those samples

^c^When the *TMEM240* expression level in breast tumors was less than half of the mean of *TMEM240* expression levels in adjacent normal breast tissues was defined as low expression

^d^The *TMEM240* promoter methylation level in breast tumors being twofold higher than in adjacent normal breast tissues was defined as hypermethylation

### Promoter hypermethylation and low *TMEM240* mRNA expression in Taiwanese breast cancer patients

Low expression of TMEM240 protein was observed in breast cancer patients. We investigated whether *TMEM240* mRNA was also expressed at lower levels in breast cancer. We analyzed *TMEM240* mRNA expression in 52 paired Taiwanese breast cancer tissues. In 50.0% (26/52) of these tissues, *TMEM240* mRNA expression was lower in the breast cancer tumor tissue than in the normal breast tissue (Fig. 3D; Table 1). We further analyzed the methylation patterns of *TMEM240* in paired 101 Taiwan breast cancer patients, the methylation level of TMEM240 was at least twofold higher in 54.5% (55/101) breast tumor tissues than in the matched normal breast tissues (Fig. 3E, F; Table 1). The DNA hypermethylation levels and mRNA expression levels of *TMEM240* showed a significant negative correlation by Spearman rank correlation coefficient analysis (*p* = 0.037). To determine whether hypermethylation of *TMEM240* is involve in the regulation of mRNA expression, *TMEM240* mRNA expression was investigated using administration of the DNA demethylating drug decitabine (DAC) to T47D and MDA-MB-231 breast cancer cells. The cells were treated with DMSO and DAC for 48 h. In the DAC groups for the two cell lines, methylation of *TMEM240* decreased to 28.36% and 7.8%, respectively, of that in the DMSO group (*p* = 0.001, Fig. 3G, H, left panel), and *TMEM240* mRNA expression increased by 150-fold and 337-fold, respectively (*p* < 0.001, Fig. 3G, H, right panel), suggesting that hypermethylation of the *TMEM240* promoter is the main mechanism through which *TMEM240* silencing occurs.

### *TMEM240* promoter hypermethylation and low mRNA expression in breast cancer tissues from the TCGA dataset

**Fig. 4:**
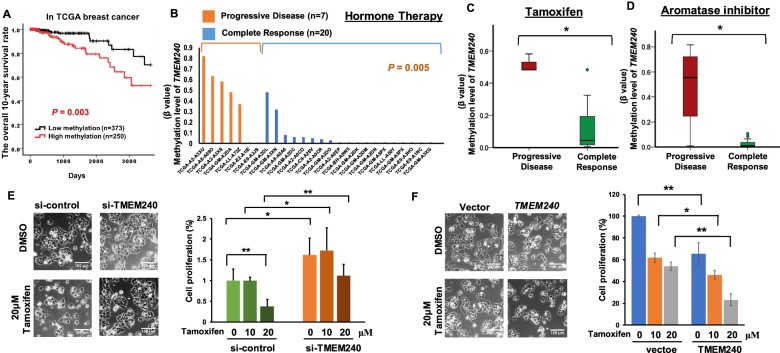
Hypermethylation of *TMEM240* is associated with poor hormone therapy response and poor survival in breast cancer. **A** Kaplan–Meier survival curves were used to compare 10-year survival in breast cancer patients with low and high *TMEM240* promoter hypermethylation. **B** Bar charts showing *TMEM240* methylation levels in breast cancer patients. Orange bar: breast cancer patients with progressive disease after hormone therapy; blue bar, breast cancer patients with complete response after hormone therapy. **C**, **D** Box plots of *TMEM240* methylation levels in patients with complete response or progressive disease after tamoxifen treatment (**C**) or aromatase inhibitor treatment (**D**). **E**, **F** Cell proliferation assays were performed in T47D cells treated with tamoxifen after si-*TMEM240* transfection (**E**, right) or pMyc-DDK-h*TMEM240* plasmid transfection (**F**, right). Brightfield views (**E**–**F**, left) are presented to illustrate the cell morphology

To further evaluate alterations in *TMEM240* hypermethylation and mRNA expression in Western breast cancer patients, we analyzed the TCGA data of the Illumina Infinium HumanMethylation450 BeadChip array for 78 breast cancer tumors, 78 matched normal tissues and 623 breast cancer tumor tissues and displayed the methylation levels in a heatmap. The exon 1 region of *TMEM240* was hypermethylated in 40.3% (251/623) of the breast tumor tissues (Table 2). Analysis of RNA sequencing data from TCGA showed that *TMEM240* mRNA expression was reduced by half in 51.4% (37/72) of the breast cancer tumor tissues compared with the matched normal breast tissues (*p* = 0.019, Additional file 1: Fig. S4) and in 60.2% (458/761) of tumors from breast cancer patients (Table 2). The DNA hypermethylation levels and mRNA expression levels of *TMEM240* showed a significant negative correlation on Spearman rank correlation coefficient analysis (*p* = 0.049). Hypermethylation of *TMEM240* was associated with Asian, ER-negative, PR-negative and triple-negative breast cancer patients and patients with invasive ductal carcinoma (all *p* < 0.001, Table 2). In addition, Kaplan–Meier curves indicated that patients with hypermethylation of *TMEM240* had a poor survival rate (Fig. 4A, log rank test, *p* = 0.003). A Cox proportional hazards survival analysis further adjusted for race, age, tumor type, tumor stage and menopausal state showed that *TMEM240* promoter hypermethylation was significantly and independently associated with 10-year overall survival (Table 3, *p* = 0.002).

**Table 2:** *TMEM240* mRNA expression and promoter hypermethylation in relation to the clinical parameters of breast cancer from TCGA^a^

Characteristics	Total	TMEM240 mRNA^b^				TMEM240 methylation			
		Low n (%)		High n (%)		Low n(%)		High n(%)	
Overall	714	427 (59.8)		287 (40.2)	582	351 (60.3)		231 (39.7)	
Age	761								
< 65	551	331 (60.1)		220 (39.9)	453	264 (58.3)		189 (141.7)	
≥65	210	127 (60.5)		83 (39.5)	170	108 (63.5)		251 (40.3)	
Race	525								
White	421	259 (61.5)		162 (38.5)	338	234 (69.2)		104 (30.8)	
Black/African American	72	41 (56.9)		31 (43.1)	64	19 (29.7)		45 (70.3)	
Asian	32	27 (84.4)		5 (15.6)^0.022^	31	14 (45.2)		17 (54.8) ^<0.001^	
Menopause state	480								
Premenopause	123	76 (61.8)		47 (38.2)	103	69 (67.0)		34 (33.0)	
Perimenopause	16	9 (56.3)		7 (43.8)	14	6 (42.9)		8 (57.1)	
Postmenopause	341	218 (63.9)		123 (36.1)	287	174 (60.6)		113 (39.4)	
Histological type	714								
ILC	170	65 (38.2)		105 (61.8)	152	113 (74.3)		39 (25.7)	
IDC	504	341 (67.7)		163 (32.3)^<0.001^	400	215 (58.8)		185 (46.3)^<0.001^	
Mucinous carcinoma	15	8 (53.3)		7 (46.7)	14	11 (78.6)		3 (21.4)	
Mixed type	25	13 (52.0)		12 (48.0)	16	12 (75.0)		4 (25.0)	
Tumor stage	535								
I and II	385	247 (64.2)		138 (35.8)	311	189 (60.8)		122 (39.2)	
III and IV	150	86 (57.3)		64 (42.7)	129	80 (62.0)		49 (38.0)	
Tumor Size	539								
T0–T1	137	93 (67.9)		44 (32.1)	114	80 (70.2)		34 (29.8)	
T2–T4	402	243 (60.4)		159 (39.6)	329	191 (58.1)		138 (41.9)^0.022^	
ER	535								
Negative	117	84 (71.8)		33 (28.2)^0.023^	103	22 (21.4)		81 (78.6)^<0.001^	
Positive	418	252 (60.3)		166 (39.7)	340	49 (73.2)		91 (26.8)	
PR	533								
Negative	169	118 (69.8)		51 (30.2)^0.020^	143	45 (31.5)		98 (68.5)^<0.001^	
Positive	364	216 (59.3)		148 (40.7)	299	225 (75.3)		74 (24.7)	
HER2	463								
Negative	404	245 (60.6)		159 (39.4)	340	220 (64.7)		120 (35.3)	
Positive	59	41 (69.5)		18 (30.5)	45	20 (44.4)		25 (55.6)^0.008^	
TNBC	515								
Yes	76	54 (71.1)		22 (28.9)	68	13 (19.1)		55 (80.9)^<0.001^	
No	439	267 (60.8)		172 (39.2)	358	255 (71.2)		103 (28.8)	

^a^These results were analyzed by the Pearson *X*^*2*^ test. *P* values with significance are shown as superscripts

^b^When the *TMEM240* expression level in breast tumors was less than half of the mean of TMEM240 expression levels in adjacent normal breast tissues was defined as low expression from TCGA data set using RNA sequencing analysis

**Table 3:** Cox proportional hazard model of clinical parameters and *TMEM240* DNA methylation level associated with breast cancer

Breast cancer	10-year overall survival^a^					
	Univariate analysis			Multivariate analysis		
Variable	HR	95% CI	*P*-value	HR	95% CI	*P*-value
Race	0.902	0.568–1.433	0.662	1.389	0.553–3.489	0.484
Age	2.055	1.366–3.091	0.001***	1.516	0.508–4.529	0.456
Tumor type	1.391	1.028–1.882	0.033*	1.241	0.567–2.716	0.588
Stage	2.052	1.311–3.213	0.002**	4.098	1.551–10.827	0.004***
Menopause	1.256	0.904–1.746	0.174	1.498	0.787–2.815	0.221
*TMEM240*	2.747	1.364–5.531	0.005**	6.172	1.984–19.197	0.002**

^a^These results were analyzed by the Cox regression model

^b^The *TMEM240* DNA methylation levels were derived from 640 breast cancer patients in TCGA data set

^c^*P < 0.05; **P < 0.01; ***P < 0.001

### Hypermethylation of *TMEM240* in breast cancer was associated with poor treatment response in the TCGA cohort set

To further investigate whether hypermethylation of *TMEM240* is associated with poor treatment response, we analyzed the relationship between hypermethylation of *TMEM240* and clinical treatment response to chemotherapy, hormone therapy and targeted therapy in patients from the TCGA cohort. The results indicated that patients with hypermethylation of *TMEM240* had poor chemotherapy response (Table 4, *p* = 0.012) and poor hormone therapy response (Table 4, *p* < 0.001). Better hormone therapy response was observed in 85.0% of patients with lower methylation of *TMEM240* but in only 28.6% of patients with hypermethylation of *TMEM240* (Table 4B, Mann–Whitney U test, *p* = 0.005). Higher methylation of *TMEM240* was associated with poorer response to tamoxifen treatment (Fig. 4C, Mann–Whitney U test, *p* = 0.041) and with poorer response to aromatase inhibitor treatment (Fig. 4D, Mann–Whitney U test, *p* = 0.037).

**Table 4:** *TMEM240* promoter hypermethylation in relation to drug treatment response in TCGA cohort^a^

Characteristics	Total	Complete response N (%)	Progressive disease N (%)	*P* value
Chemotherapy^b^	257	239 (93.0)	18 (7.0)	
Low methylation	99	97 (98.0)	2 (2.0)	0.012
High methylation	158	142 (89.9)	16 (10.1)	
Hormone therapy^c^				
Low methylation	24	21 (87.5)	3 (12.5)	< 0.001
High methylation	11	2 (18.2)	9 (81.8)	
Targeted molecular therapy^d^				
Low methylation	4	4 (100.0)	0 (0.0)	0.515
High methylation	8	6 (75.0)	2 (25.0)	

^a^These results were analyzed by the Fisher’s exact test. The patients with a treatment duration of greater than 4 weeks were included in this analysis. When the β value of *TMEM240* methylation level in breast tumors was higher than 0.25 was defined as hypermethylation from TCGA data set using Infinium Human Methylation 450 K BeadChip

^b^Chemotherapy drugs:

antimetabolites drugs: 5-fluorouracil, capecitabine, gemcitabine, methotrexate

alkylating drugs: cyclophosphamide, cisplatin and carboplatin

topoisomerase inhibitors: doxorubicin, mitoxantrone and epirubicin

microtubule inhibitors: taxanes, vinca alkaloids, and epothilones

^c^Hormone therapy drug:

Estrogen inhibitors: tamoxifen and fulvestrant

aromatase inhibitors: letrozole, anastrozole and exemestane

^d^Targeted Molecular therapy: Avastin and Herceptin

### High TMEM240 expression enhance the tamoxifen treatment response in breast cancer cell lines

To further investigate whether the expression of *TMEM240* may be involved in the response to hormone drug treatment, a cell proliferation assay was performed after overexpression and/or knockdown of *TMEM240* and tamoxifen treatment in T47D breast cancer cells. The proliferation of T47D (ER + /PR +) cells was significantly decreased by 62.9% when cells transfected with si-control were treated with 20 µM tamoxifen (*p* = 0.003), but only a 31.1% decrease in proliferation was observed in cancer cells transfected with si-*TMEM240* (Fig. 4E). The data indicate that lower expression of TMEM240 is related to resistance to tamoxifen treatment. Overexpression of TMEM240 in T47D cells induced 76.9% cancer cell death when the cells were treated with 20 µM tamoxifen but only a 46.0% decrease in the vector control cancer cells when treated with 20 µM tamoxifen (Fig. 4F).

### Circulating methylated *TMEM240* predicts disease progression and poor hormone therapy response in Taiwanese breast cancer patients

**Fig. 5:**
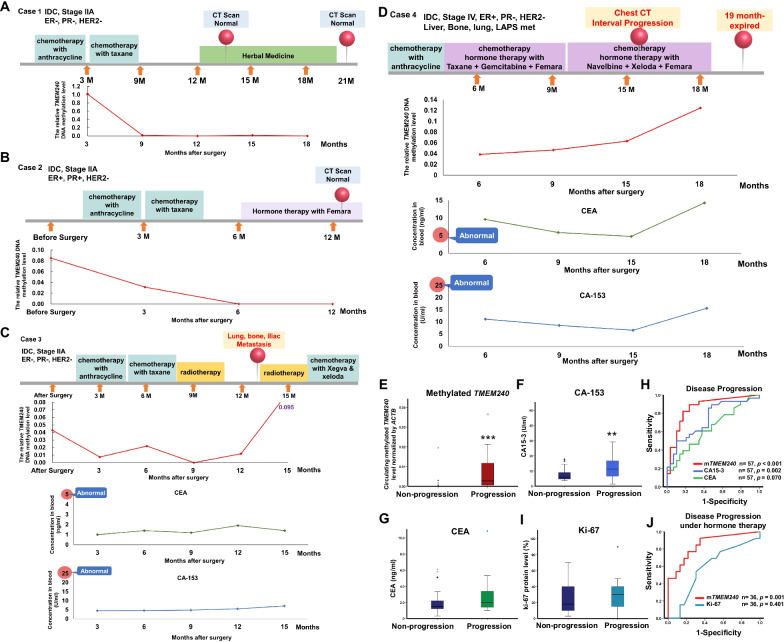
Circulating methylated *TMEM240* is increased in Taiwanese breast cancer patients with disease progression and poor hormone therapy response. The level of circulating methylated *TMEM240* dramatically and gradually decreased in triple-negative breast cancer patients (**A**) and in breast cancer patients who received hormone therapy and did not experience recurrence or metastasis (**B**). **C** Recurrence and metastasis were detected in triple-negative breast cancer patients when circulating methylated *TMEM240* was found in such patients after treatment, and it thereafter increased again. The concentrations of CEA and CA15-3 in serum remained normal. **D** The level of circulating methylated *TMEM240* gradually increased in breast cancer patients with disease progression even after hormone therapy and chemotherapy. The concentrations of CEA and CA15-3 in serum are incapable of early monitoring of disease progression and poor treatment response. **E** Box plot showing the levels of circulating methylated *TMEM240* in the plasma of 32 patients with recurrence/metastasis and in the plasma of 29 patients without recurrence/metastasis. **F**, **G** Box plots showing the concentrations of CEA and CA15-3 in the sera of 28 patients with recurrence/metastasis and in the sera of 29 patients without recurrence/metastasis. **P < 0.01; ***P < 0.001. **H** ROC curves for disease progression prediction were calculated using the measured circulating methylated *TMEM240* levels and the measured concentrations of CEA and CA15-3. **I** A box plot of the percentage of Ki-67 expression level in breast cancer tumors from 13 patients with recurrence/metastasis and in tumors from 23 patients without recurrence/metastasis is shown. **J** ROC curves for disease progression and hormone therapy response prediction were calculated using circulating methylated *TMEM240* levels and Ki-67 expression levels

Hypermethylation of *TMEM240* was found in breast tumors of patients who displayed poor treatment response, especially in tumors from patients who received hormone therapy. Detection of circulating methylated *TMEM240* in the plasma of patients with poor treatment response could provide a potential tool for real-time monitoring of clinical outcomes after medical treatment. Breast cancer patients were recruited from the Taipei Medical University Hospital and Shuang Ho Hospital and were followed up for at least 1 year. After these patients received treatment, circulating methylated DNA was extracted from their plasma at 3–6 month intervals and analyzed by QMSP. The patients with poor prognosis had significantly higher circulating methylated *TMEM240* levels than other patients but did not display higher levels of CA-15-3 and CEA (Table 5). The level of circulating methylated *TMEM240* dramatically and gradually decreased in breast cancer patients following treatment (Case 1 and Case 2, Fig. 5A, B). When patients experienced disease progression, recurrence or metastasis, the levels of circulating methylated *TMEM240* increased significantly (Case 3 and Case 4, Fig. 5C–E, Mann–Whitney U test, *p* < 0.001). The circulating methylated *TMEM240* test for poor prognosis prediction was found to have 87.5% sensitivity (28/32), 93.1% specificity (27/29), and 90.2% accuracy (55/61), values that are better than those obtained using the currently used biomarkers CEA and CA-153 (Table 5; Fig. 5E–H).

**Table 5:** *TMEM240* promoter hypermethylation in relation to prognosis and drug treatment response in plasma of Taiwanese breast cancer patients^a^

Characteristics	Total^b^	Non-progression N (%)	Progressive^c^ disease N (%)	*P* value
Overall	61	29 (47.5)	32 (52.5)	
*TMEM240* in plasma				
No methylation	31	27 (87.1)	4 (12.9)	< 0.001
High methylation	30	2 (6.7)	28 (93.3)	
CA-153 in serum				
Normal	55	29 (52.7)	26 (47.3)	0.237
Abnormal (> 25 units/mL)	2	0 (33.3)	2 (100.0)	
CEA in serum				
Normal	49	26 (53.1)	23 (46.9)	0.470
Abnormal (> 5 ng/mL)	9	3 (33.3)	6 (66.7)	
Underwent hormone therapy	36	23 (63.9)	13 (36.1)	
*TMEM240* in plasma				
Low methylation	25	22 (88.0)	3 (12.0)	< 0.001
High methylation	11	1 (9.1)	10 (90.9)	
Ki-67 in breast tumors				
Low expression	16	10 (62.5)	6 (37.5)	1.000
High expression (> 15%)	20	13 (65.0)	7 (35.0)	

^a^These results were analyzed by the Fisher’s exact test. The patients with a treatment and monitoring duration of greater than one year were included in this analysis. When the circulating methylated *TMEM240* levels normalized by circulating *ACTB* in plasma of breast cancer patients was higher than 0.002 was defined as abnormal

^b^For concentration of CA-153, CEA and Ki-67 expression, the number of samples (n) was lower than the overall number analyzed because clinical data were unavailable for those samples

^c^Non-progression: patients without progression, recurrence, metastasis

Progressive disease: patients with progression, recurrence, metastasis

Hypermethylation of *TMEM240* in breast cancer was associated with poor response to hormone therapy in the TCGA cohort. We further investigated whether patients with an increase in circulating methylated *TMEM240* in plasma experienced disease progression, recurrence or metastasis after hormone therapy. The results indicated that the patients with poor response after hormone therapy had significantly higher levels of circulating methylated *TMEM240* (Table 5). The circulating methylated *TMEM240* test for poor hormone therapy response prediction was found to have a sensitivity of 76.9% (10/13), a specificity of 95.7% (22/23), and an accuracy of 88.9% (32/36) (Fig. 5E, I, J).

### Pathways and networks of TMEM240 involved in estradiol metabolism and cancer progression

**Fig. 6:**
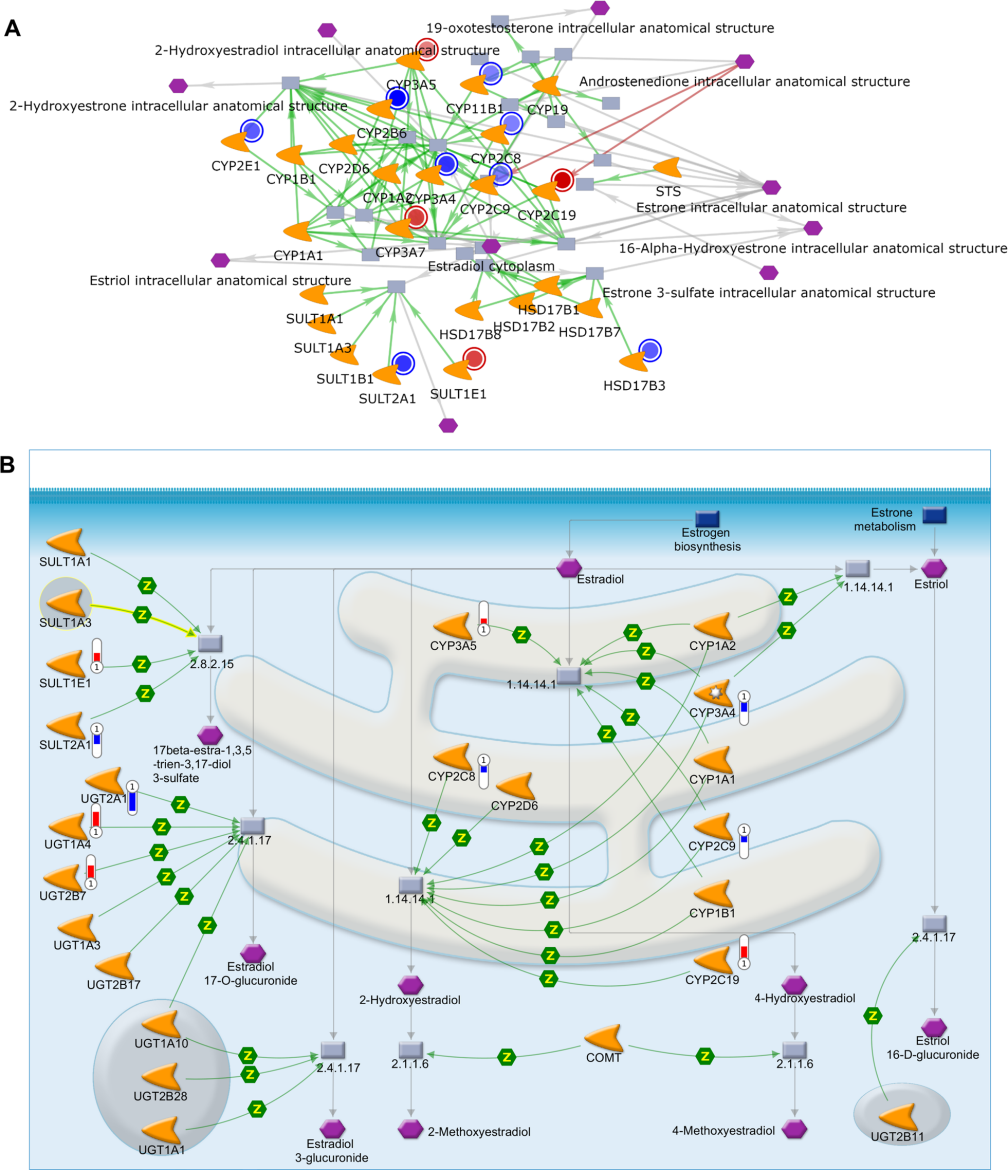
Network analysis of TMEM240 involved in estrone and estradiol metabolism. MetaCore Metabolic Networks (Endogenous) analysis found that TMEM240 was involved in the metabolic networks of steroid, estrone, and estradiol metabolism (**A**). TMEM240, which is involved in estradiol metabolism, was identified with MetaCore Pathway Maps (**B**). RNA-seq indicated decreases in CYP2B6, CYP11B1, CYP2C8, CYP2C9, CYP3A4, CYP2E1, HSD17B3, SULT2A1, SULT1B1, and UGT2A1 mRNA expression and increases in CYP2C19, CYP3A5, CYP3A7, SULT1E1, UGT1A4, and UGT2B7 mRNA expression under TMEM240 overexpression compared to the vector control. The enzymes are responsible for Androstenedione, Estrone, Estradiol, and Estriol metabolism processes

To analyze what pathways mediated by TMEM240 are associated with the repression of breast cancer progression, exogenous TMEM240 expression was enforced in MDA-MB-231 cells, and RNA-seq and MetaCore were used for data analysis. The results indicated that TMEM240 decreased the expression levels of fibroblast growth Factor 2 (FGF2), stromal cell-derived factor-1 (SDF-1), vav guanine nucleotide exchange Factor 1 (VAV-1), C–C motif chemokine receptor 2 (CCR2), nuclear factor-κB (NF-kB), oncostatin M, and matrix metalloproteinase-2 (MMP2), which stimulate cell proliferation, migration, and the cancer epithelial–mesenchymal transition (EMT) process, and increased the expression levels of E-cadherin (CDH1) and vascular endothelial (VE)-cadherin, which inhibit cell migration (Additional file 1: Figs. S5 and S6). TMEM240 overexpression also regulated several enzymes involved in estradiol metabolism. Overexpression of TMEM240 altered the expression levels of enzymes involved in estrone and estradiol metabolism, including cytochrome P450 (CYP) 2B6 (CYP2B6), CYP11B1, CYP2C8, CYP2C9, CYP2C19, CYP2E1, CYP3A4, CYP3A5, CYP3A7, hydroxysteroid 17-beta dehydrogenase 3 (HSD17B3), sulfotransferase family (SULT) 2A member 1 (SULT2A1), SULT1B1 SULT1E1, UDP glucuronosyltransferase family 1 member A4 (UGT1A4), UGT2B7, and UGT2A1 (Fig. 6). The data have been deposited with links to BioProject accession number PRJNA804313 in the NCBI database (https://www.ncbi.nlm.nih.gov/bioproject/).

## Discussion

Aberrant promoter hypermethylation of CpG islands associated with TSGs can cause transcriptional silencing and contribute to tumorigenesis. In the present investigation, hypermethylation of *TMEM240* in patients with poor hormone therapy response was identified using genome-wide methylation array analysis. QMSP confirmed the presence of *TMEM240* hypermethylation in Taiwanese breast cancer tumor tissues compared with normal tissues. In the TCGA cohort, hypermethylation of the promoter region of *TMEM240* was found in 40.3% of tumors. It occurs more frequently in Asian patients (54.8%). Results similar to those found in the Asian TCGA cohort were consistently found in Taiwanese breast cancer patients (54.5%). Low expression of TMEM240 protein was found in most Taiwanese and Korean breast cancer patients. No local distribution of CNVs in the *TMEM240* gene was found in breast tumors from patients. The results suggest that TMEM240 mRNA and protein expression deficiency was primarily mediated by hypermethylation of the *TMEM240* gene.

Moreover, patients with hypermethylation of *TMEM240* had poor 10-year overall survival. hypermethylation of *TMEM240* was observed in patients with progressive disease, especially in patients treated with hormone therapy. The results obtained for Taiwanese breast cancer patients were similar to those obtained for patients in the TCGA cohort. Hypermethylation of *TMEM240* in 87.8% of CRC, 80.0% of esophageal cancer and 80.4% of liver cancer patients was reported in our previous study (Chang et al. 2020). Here, we further analyzed a TCGA cohort and found high methylation of *TMEM240* in breast cancer and in endometrial and uterine cancer. Alterations in *TMEM240* in female cancer are less frequent than those that occur in gastrointestinal cancer, but they are associated with poor clinical treatment response and poor prognosis in breast cancer. Circulating methylated *TMEM240* dramatically and gradually decreased and then diminished in blood of breast cancer patients without disease progression, whereas it increased in blood of breast cancer patients with recurrence or metastasis. Hypermethylation of *TMEM240* leads to low expression of *TMEM240* mRNA and low TMEM240 protein expression. The membrane protein TMEM240 also negatively regulated several extracellular and membrane proteins involved in cell proliferation, migration, and cancer EMT, such as SDF-1, FGF2, oncostatin M, and MMP2 (Additional file 1: Fig. S5). The transfection factors downstream of the membrane protein TMEM240 involved in extracellular and membrane protein expression are worthy of further investigation. TMEM240 expression induces breast cancer cell death and enhances the cellular response to hormone therapy drugs, suggesting that deficiency in TMEM240 expression plays an important role during cancer progression in breast cancer patients.

In addition to its association with progressive disease and poor prognosis, hypermethylated *TMEM240* was found to be strongly associated with ER/PR negative breast cancer, TNBC and poor hormone therapy response. Patients with hypermethylation of *TMEM240* often had poor hormone therapy response (Table 3, *p* < 0.001) (Fig. 5). Even patients with hypermethylation of *TMEM240* and positive ER/PR expression exhibited poor hormone therapy responses, including treatment with tamoxifen or aromatase inhibitors (AIs) (Table 4). Only in patients in which TMEM240 was expressed at sufficient levels in the cancer cells did hormone therapy produce a good therapeutic response (Figs. 2, 3). Biomarkers based on DNA methylation could potentially be applied for treatment decisions (Jovanovic et al. 2010). Ki-67 is a parameter, and multigene analysis (MGA) has been used to predict the response to hormone therapy (Untch et al. 2019; Yerushalmi et al. 2010). The DNA methylation status of *CYP1B1* has been reported to potentially be a marker for treatment response in patients receiving and not receiving tamoxifen as a hormonal treatment (Widschwendter et al. 2004). Our data found that TMEM240 regulates the expression of a dozen enzymes involved in estrone and estradiol metabolism, including estradiol-metabolizing cytochrome p450 enzymes. The data indicate that TMEM240 deficiency is involved in a poor hormone treatment response and may be mediated by interfering with estrogen biosynthesis and metabolism. Methylation of *TMEM240* may play a role in determining treatment response to hormone therapy drugs.

Almost all triple-negative breast cancer patients (95.7%, 22/23) displayed deficient TMEM240 protein expression. Patients who had circulating hypermethylated *TMEM240* experienced disease progression (Fig. 5). TNBC represents a group of breast cancers with heterogeneous genomic features. There are several different subtypes of TNBC, including the Vanderbilt subtype and the Baylor subtype (Lehmann et al. 2011; Ahn et al. 2016). Each subtype carries a different set of mutant genes (Lehmann et al. 2011; Ahn et al. 2016). Further study may focus on the relationship between TMEM240 and specific subtypes of TNBC.

Advances in detection technology have reduced breast cancer death rates in several Western countries (DeSantis et al. 2019). Therefore, the development and use of biomarkers of treatment response can improve patient outcomes. The presence of breast-derived circulating DNA is indicative of residual disease after treatment (Moss et al. 2020). Circulating methylated *TMEM240* dramatically and gradually decreases and then diminishes in patients with various subtypes of breast cancer who do not show disease progression (Fig. 5), suggesting that measurement of circulating methylated *TMEM240* could be used to detect the presence of residual disease. In addition, the level of circulating methylated *TMEM240* in plasma increased further in breast cancer patients with recurrence or metastasis (Fig. 5). In these patients, the concentrations of CEA and CA15-3 in serum remained normal or increased much later than did the circulating methylated *TMEM240*. The detection of CEA and CA15-3 was incapable of revealing disease progression and poor treatment response in several patients (Table 5). Measurement of circulating methylated *TMEM240* could be used to monitor and detect early disease progression after treatment and during long-term follow-up. Although hypermethylation of *TMEM240* also occurs in other types of cancer, its high alteration in cancers may assist the detection of disease progression. Combining measurement of *TMEM240* hypermethylation with the measurement of additional breast cancer-specific methylated DNA biomarkers that are associated with disease progression will improve detection sensitivity and cancer specificity.

## Conclusions

Deficiency in TMEM240 expression plays an important role during cancer progression in breast cancer patients. Circulating hypermethylated *TMEM240* may represent a potential biomarker for disease progression and poor hormone therapy response.

## Supplementary Information

**Additional file 1: Table S1. **List of primers sequences and their reaction conditions used in the present study. Table S2 List of antibodies used in the present study.** Figure S1. **Representative standard sequencing diagram for bisulfite direct sequencing of the *TMEM240* gene. Figure S2 Copy number variation (CNV) analysis and chromosomal distribution of localized CNVs. (A) The workflow of CNV analysis. (B) CNV gain is shown as a red peak. (C) CNV loss is shown in the blue peak. **Figure S3. **The cell morphology was determined by microscopy in MDA-MB-231 cells (original magnification, ×100). **Figure S4. **Representative figures showing the *TMEM240* mRNA expression by RNA sequencing in breast cancer patients from TCGA. **Figure S5**. Pathway Maps analysis of TMEM240 involvement in the epithelial–mesenchymal transition (EMT) process. MetaCore Pathway Maps analysis indicated that TMEM240 expression led to decreases in FGF2, NFkB, MMP2, and Oncostatin M and increases in E-cedherin and VE-cadherin. **Figure S6**. Pathway maps analysis of TMEM240 involvement in the SDF-1 pathway. MetaCore pathway maps analysis indicated that TMEM240 expression led to decreases in SDF-1, G-protein alpha-i2, and VAV-1 expression.

## Data Availability

The data generated in this study are available from the corresponding author upon reasonable request.

## Abbreviations

ACTB: Beta-actin
BC: Breast cancer
CA15-3: Cancer antigen 15-3
ccfDNA: Circulating cell-free DNA
CCR2: C–C motif chemokine receptor 2
CDH1: E-cadherin
CEA: Carcinoembryonic antigen
CNV: Copy number variation
cmDNA: Circulating methylated DNA
CYP: Cytochrome P450 family
EMT: Epithelial–mesenchymal transition
ESR1: Estrogen receptor 1
FGF2: Fibroblast growth Factor 2
GAPDH: Glyceraldehyde 3-phosphate dehydrogenase gene
GDC: Genomic Data Commons
HSD17B3: Hydroxysteroid 17-beta dehydrogenase 3
Ki-67: Marker of proliferation Ki-67
MMP2: Matrix metalloproteinase-2
NF-kB: Nuclear factor-κB
QMSP: Quantitative methylation specific real-time polymerase chain reaction
QPCR: Quantitative real-time reverse transcription polymerase chain reaction
QS: Overall survival
RNA-seq: RNA sequencing
SAC21: Spinocerebellar ataxia 21
SDF-1: Stromal cell-derived factor-1
SRB: Sulforhodamine B
SULT: Sulfotransferase family
TNBC: Triple negative breast cancer
TCGA: The Cancer Genome Atlas
TMEM240: Transmembrane Protein 240
TSG: Tumor suppressor gene
TMU: Taipei Medical University
UDP: Glucuronosyltransferase family
VAV-1: Vav guanine nucleotide exchange Factor 1
VE-cadherin: Vascular endothelial cadherin
