# Supplementary material for: Hypermethylation of TMEM240 predicts poor hormone therapy response and disease progression in breast cancer
Source: Mol Med. 2022 Jun 17;28:67. doi: 10.1186/s10020-022-00474-9 (PMC9204905; doi:10.1186/s10020-022-00474-9)
Supplement: Supplementary file 1 — Additional file 1: Table S1. List of primers sequences and their reaction conditions used in the present study. Table S2 List of antibodies used in the present study. Figure S1. Representative standard sequencing diagram for bisulfite direct sequencing of the TMEM240 gene. Figure S2 Copy number variation (CNV) analysis and chromosomal distribution of localized CNVs. (A) The workflow of CNV analysis. (B) CNV gain is shown as a red peak. (C) CNV loss is shown in the blue peak. Figure S3. The cell morphology was determined by microscopy in MDA-MB-231 cells (original magnification, ×100). Figure S4. Representative figures showing the TMEM240 mRNA expression by RNA sequencing in breast cancer patients from TCGA. Figure S5. Pathway Maps analysis of TMEM240 involvement in the epithelial–mesenchymal transition (EMT) process. MetaCore Pathway Maps analysis indicated that TMEM240 expression led to decreases in FGF2, NFkB, MMP2, and Oncostatin M and increases in E-cedherin and VE-cadherin. Figure S6. Pathway maps analysis of TMEM240 involvement in the SDF-1 pathway. MetaCore pathway maps analysis indicated that TMEM240 expression led to decreases in SDF-1, G-protein alpha-i2, and VAV-1 expression. [file 10020_2022_474_MOESM1_ESM.docx]

**Hypermethylation of *TMEM240* Predicts Poor Hormone Therapy Response and Disease Progression in Breast Cancer**

Ruo-Kai Lin^#1,2^, Chih-Ming Su^#3,4^, Shih-Yun Lin^1,^ Le Thi Anh Thu^1,5^,

Phui-Ly Liew^6,7^, Jian-Yu Chen^8^, Huey-En Tzeng^9, 10, 11^, Yun-Ru Liu^12^,

Tzu-Hao Chang^13^, Cheng-Yang Lee^14^, Chin-Sheng Hung *^3,4,15^

**Table S1 List of primers sequences and their reaction conditions used in the present study**

| **Gene** | **primer** | **5’→3’sequences** | **Application** | **Size**  **(bp)** | **Tm (**$\mathbf{℃}$**)** |
| --- | --- | --- | --- | --- | --- |
| *GAPDH* | Forward  Reverse | AGCCACATCGCTCAGACAC  GCCCAATACGACCAAATCC | RT-PCR  Real-time | 66 | 60 |
| *TMEM240* | Forward  Reverse | ATCGCGTGCTTGATGGAC  GGATCACGTAGTGGATATGGTG | RT-PCR  Real-time | 125 | 60 |
| *BACTIN* | Forward  Reverse  Probe | TGGTGATGGAGGAGGTTTAGTAAGT  AACCAATAAAACCTACTCCTCCCTTAA  ACCACCACCCAACACACAATAACAAACACA | MSP-M | 132 | 60 |
| *TMEM240* | Forward  Reverse  Probe | TTTTTCGTTTATTATTACGATCGAC  CGACCCCGCCCGATATCCATAA  TTTAGAATTATGAAGATTATGGTGTTC | MSP-M | 81 | 60 |

**Table S2 List of antibodies used in the present study**

| **Target** | **K.D.** | **Raised In** | **Application** | **Dilution** | **Source** | **Catalog No.** |
| --- | --- | --- | --- | --- | --- | --- |
| β*-actin* | 42 | Mouse | Western blot | 1:5000 | GeneTex  Irvine,  USA | GTX26276 |
| *DDK*  Clone: 4C5 | 20 | Mouse | Western blot  Immunofluorescence | 1:3000 | OriGene Rockville,  USA | TA50011-100 |
| *c-Myc* | 20 | Rabbit recombinant oligoclonal Ab | Western blot  Immunofluorescence | 1:50 | Invitrogen  Grand Island, USA | 710007 |
| Mouse IgG-HRP conjugate |  | Goat | Western blot | 1:5000 | GeneTex  Irvine,  USA | GTX213111-01 |
| Rabbit IgG- HRP conjugate |  | Goat | Western blot | 1:5000 | GeneTex  Irvine,  USA | GTX213110-01 |
| Mouse IgG-FITC conjugate |  | Goat | Immunofluorescence | 1:250 | Sigma-aldrich  Taiwan, R.O.C | F0257 |
| Rabbit IgG-TRITC conjugate |  | Goat | Immunofluorescence | 1:250 | Sigma-aldrich  Taiwan, R.O.C | T6778 |

**
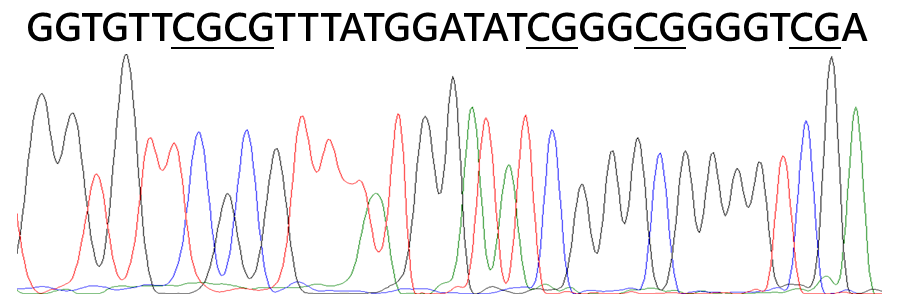
**

**Figure S1.** Representative standard sequencing diagram for bisulfite direct sequencing of the *TMEM240* gene.


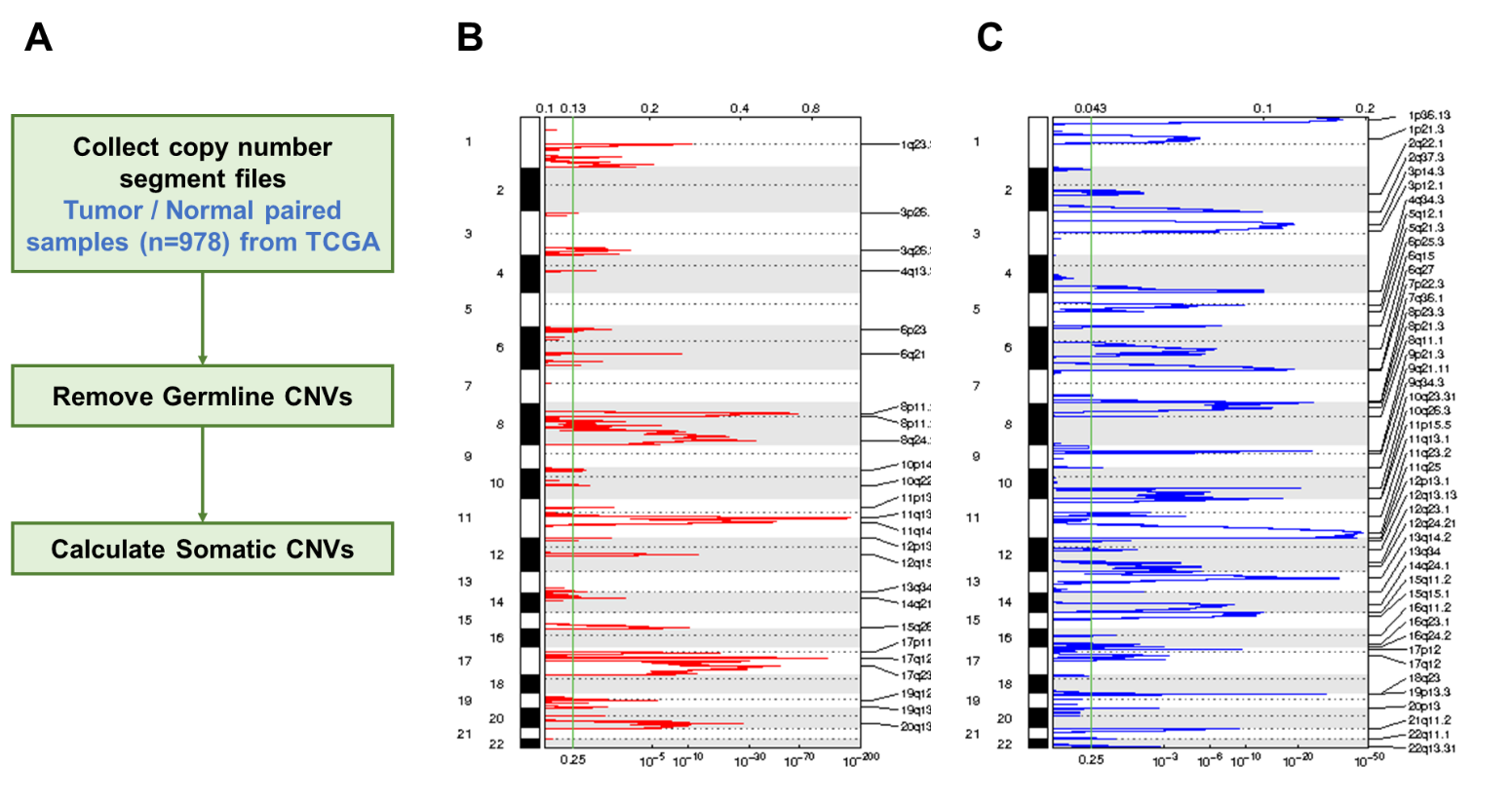


Figure S2 Copy number variation (CNV) analysis and chromosomal distribution of localized CNVs. (A) The workflow of CNV analysis. (B) CNV gain is shown as a red peak. (C) CNV loss is shown in the blue peak.


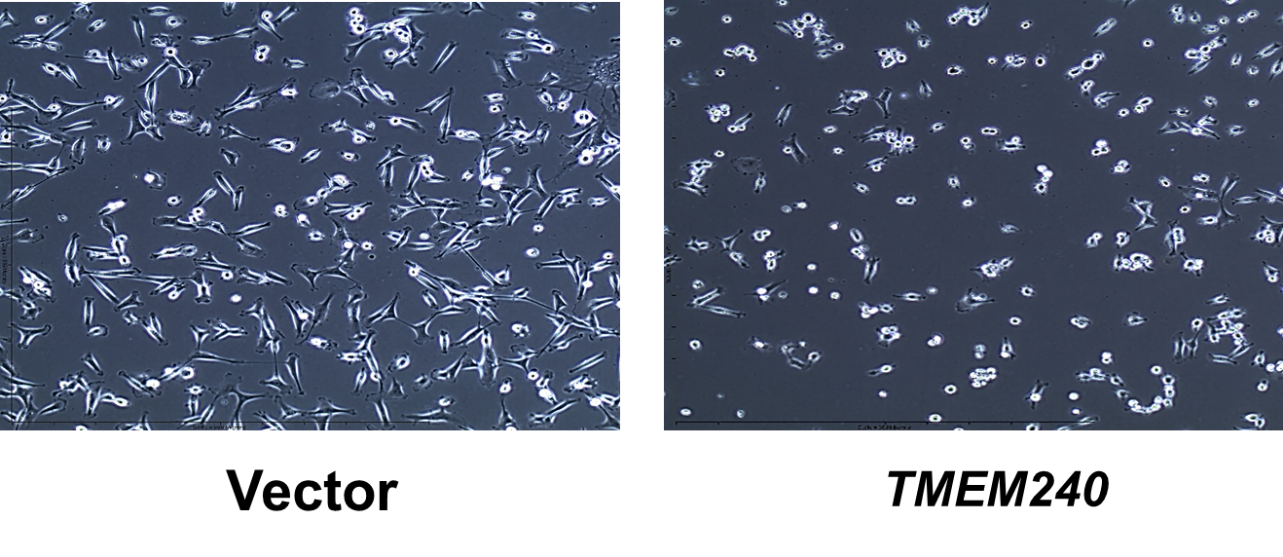


**Figure S3.** The cell morphology was determined by microscopy in MDA-MB-231 cells (original magnification, ×100).

**
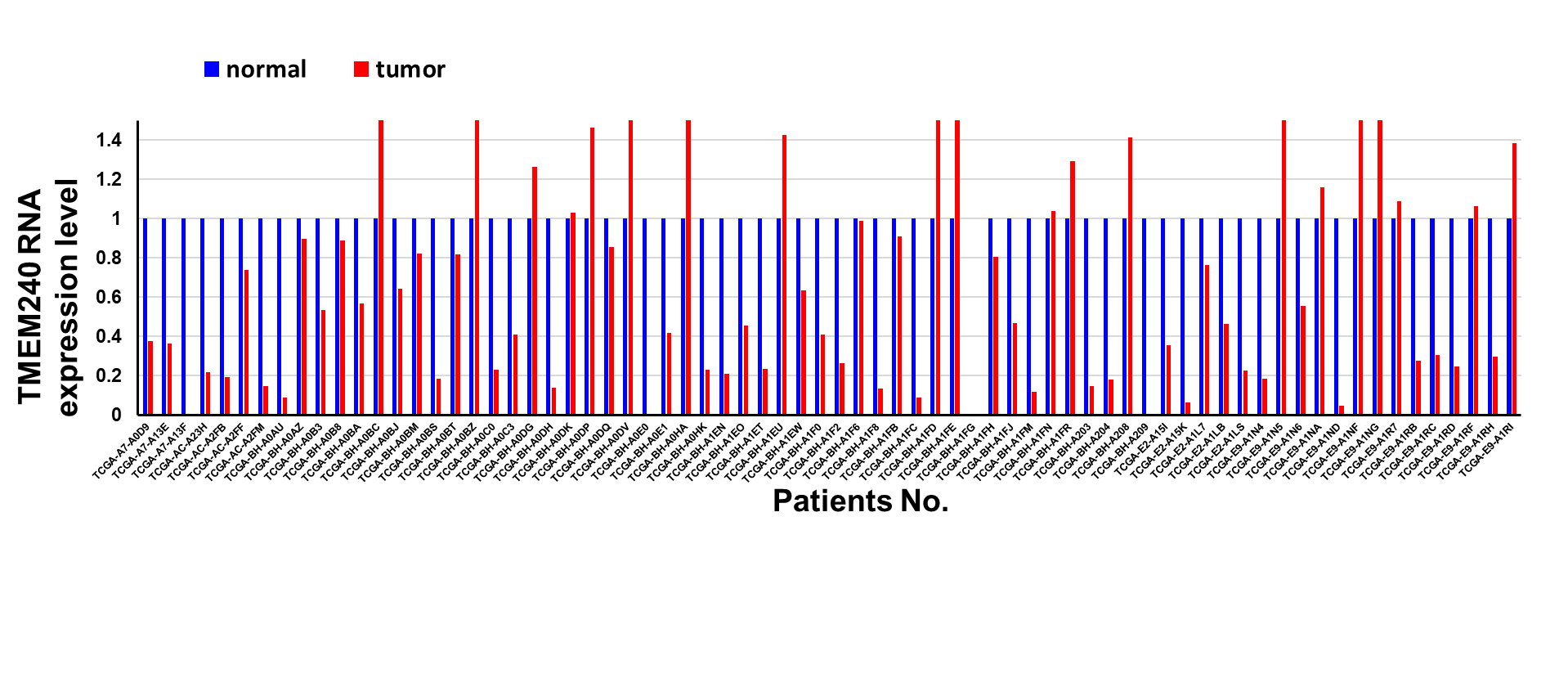
**

**Figure S4.** Representative figures showing the *TMEM240* mRNA expression by RNA sequencing in breast cancer patients from TCGA.


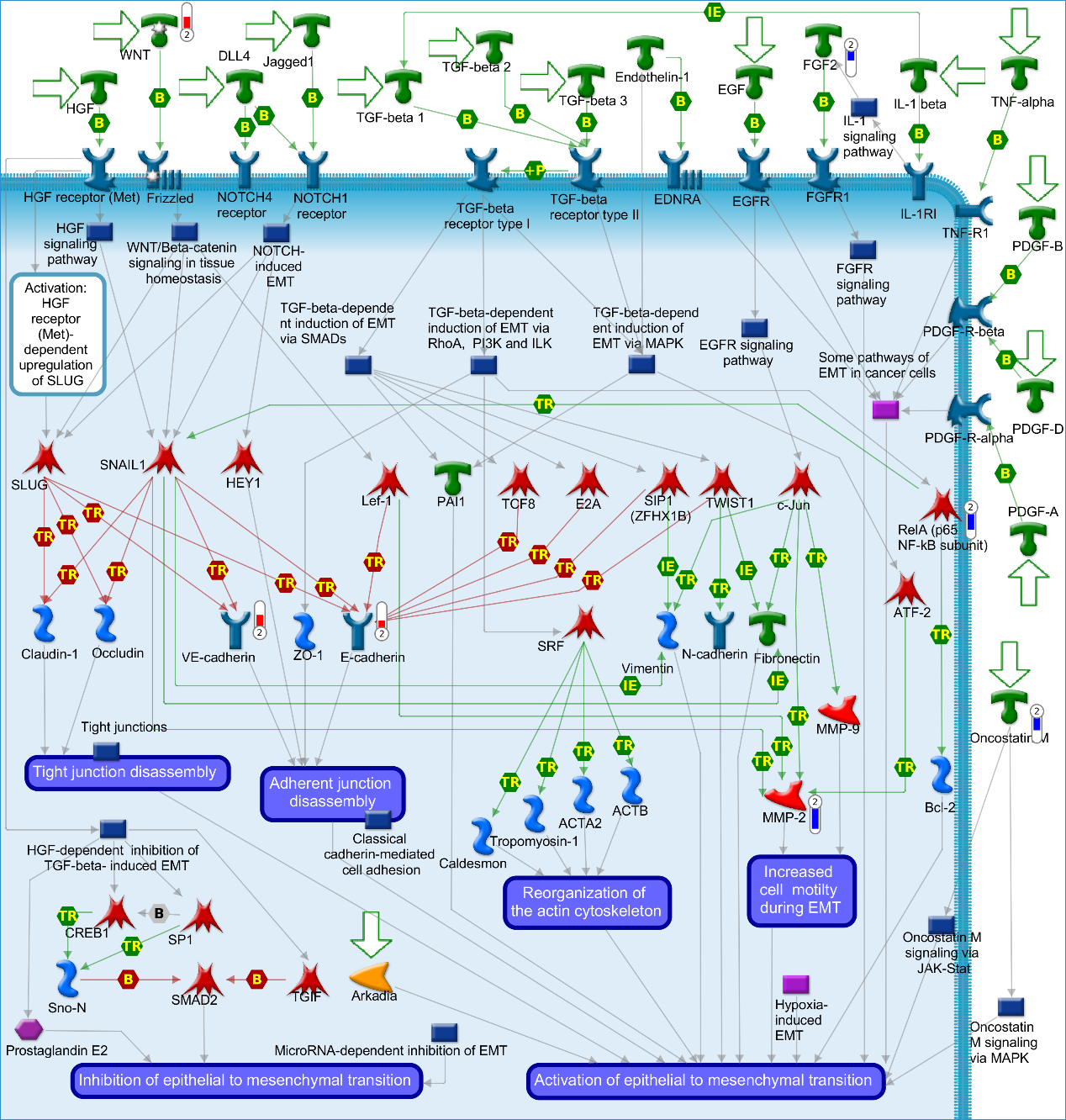


**Figure S5 Pathway Maps analysis of TMEM240 involvement in the epithelial–mesenchymal transition (EMT) process.** MetaCore Pathway Maps analysis indicated that TMEM240 expression led to decreases in FGF2, NFkB, MMP2, and Oncostatin M and increases in E-cedherin and VE-cadherin.


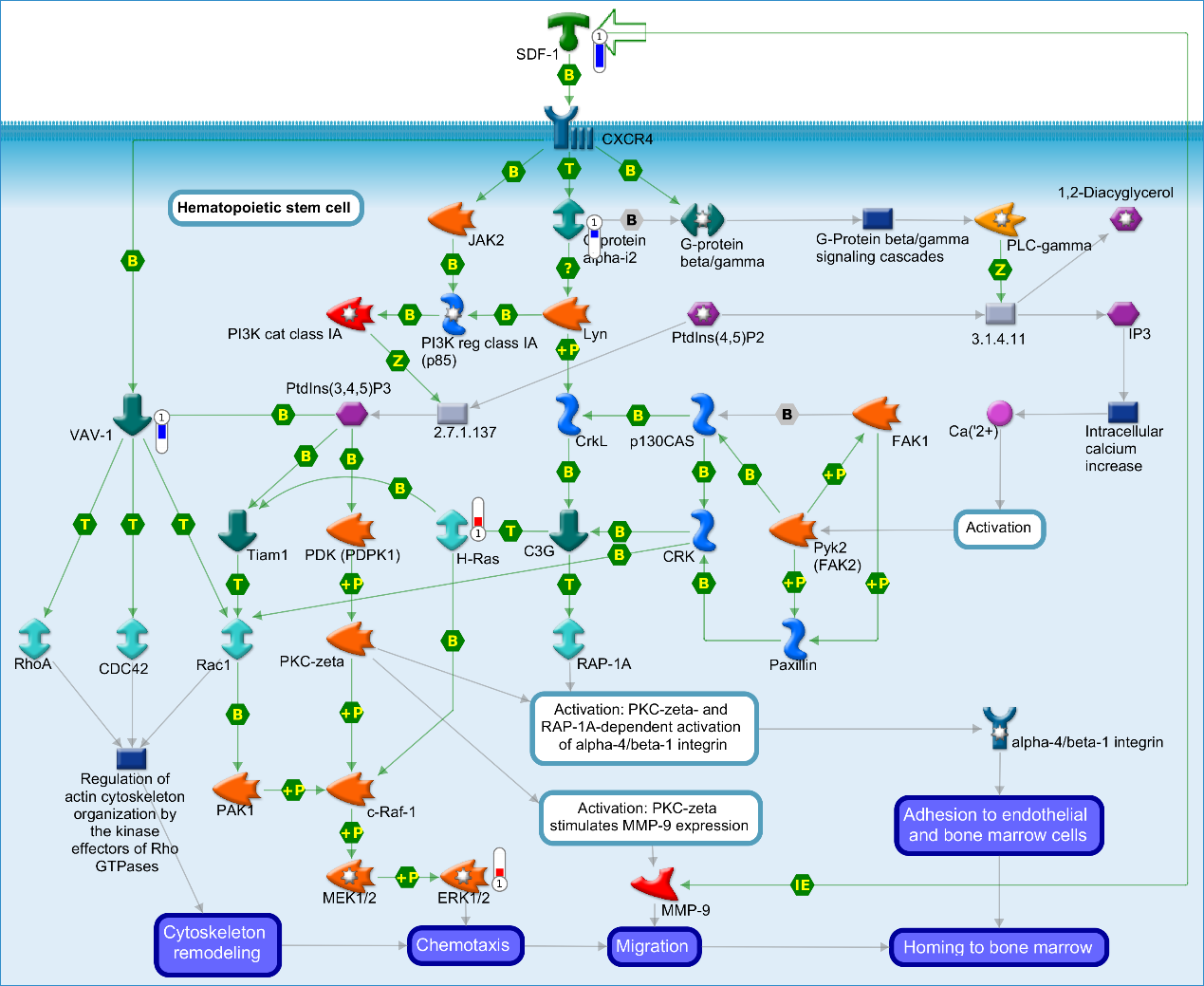


**Figure S6 Pathway maps analysis of TMEM240 involvement in the SDF-1 pathway.** MetaCore pathway maps analysis indicated that TMEM240 expression led to decreases in SDF-1, G-protein alpha-i2, and VAV-1 expression.
